# Clinical and epidemiological profile of pregnant and postpartum women affected by COVID-19 who required respiratory support

**DOI:** 10.61622/rbgo/2025rbgo14

**Published:** 2025-04-30

**Authors:** Carolina Maria Pires Cunha, Melania Maria Ramos Amorim, Julianna de Azevedo Guendler, Alex Sandro Rolland Souza, Leila Katz

**Affiliations:** 1 Instituto de Medicina Integral Prof. Fernando Figueira Recife PE Brazil Instituto de Medicina Integral Prof. Fernando Figueira, Recife, PE, Brazil.; 2 Universidade Federal de Campina Grande Campina Grande PB Brazil Universidade Federal de Campina Grande, Campina Grande, PB, Brazil.

**Keywords:** COVID-19, SARS-CoV-2, Severe acute respiratory syndrome, Pregnancy, Pregnancy trimester, third, Cesarian section, Postpartum period, Near miss, healthcare, Obesity, Intensive care units, Respiration, artificial, Noninvasive ventilation

## Abstract

**Objective:**

This study described the clinical and epidemiological profile and the management provided to pregnant and postpartum women with COVID-19 who required respiratory support.

**Methods:**

A descriptive study was conducted with pregnant and postpartum women with confirmed COVID-19 who received care between April 2020 and December 2021 in eight referral centers in northeastern Brazil. Statistical analysis was conducted using Epi-Info 7.2.5 and Medcalc, version 20.112.

**Results:**

Of the 720 patients admitted, 208 (32.7%) required respiratory support. Mean age of the participants was 28.9±7.1 years. Most (52.8%) were brown-skinned; 31.3% had little formal schooling; 41.1% had a personal income and 23.1% were married. Around half were referred from another hospital. Overall, 36.8% were obese and 36.9% were hypertensive. Criteria for severe acute respiratory syndrome (SARS) were present in 80.7% of cases. Overall, 151 patients (74.7%) required corticoids, and 150 (76.1%) were admitted to an intensive care unit. Non-invasive ventilation was needed in 89.4% of cases, with nasal catheters being the most common type (55.3% of cases). Invasive mechanical ventilation was necessary in 35.5% of cases and 91.6% had a cesarean section. Maternal near miss and death occurred in 24% and 12.9% of cases, respectively.

**Conclusion:**

Pregnant and postpartum women with COVID-19 who required respiratory support were predominantly brown-skinned, in the third trimester of pregnancy and had been referred from another hospital. The cesarean section rate was high; the presence of criteria for SARS was common and the rates of COVID-19-related maternal near miss and death were high.

**Clinical Trials registry:**

NCT04462367

## Introduction

Pregnancy leaves women more vulnerable to viral diseases such as Coronavirus Disease 2019 (COVID-19) due to physiological factors, abnormal respiratory mechanics and increased oxygen consumption, hypercoagulability and changes in the immune system.^(1)^

The COVID-19-related maternal complications and mortality have been the focus of several studies worldwide. An increased incidence of the severe forms of COVID-19 has been shown during pregnancy and in the postpartum and an association has been found between COVID-19 and adverse gestational and perinatal outcomes.^(2,3)^ In Brazil, a country where maternal mortality is habitually high, the number of deaths during pregnancy and in the postpartum has increased since the beginning of the COVID-19 pandemic.^(4)^

Although pregnant women with COVID-19 are less likely to show symptoms, they are more likely to require hospitalization, to be admitted to an Intensive Care Unit (ICU) and to require invasive ventilation, and they are at a greater risk of severe acute respiratory syndrome (SARS) compared to non-pregnant women of reproductive age.^(5)^ An increased risk of death from the disease has also been documented.^(4)^

Some studies have reported a greater risk of SARS in pregnant and postpartum women.^(6,7)^ Nevertheless, more robust data on pregnant women with COVID-19-related SARS admitted to ICU are not yet available for this particular setting. Therefore, it is important to characterize this population as well as the course of the disease, its management and outcomes, adding data to what is already known and contributing substantially to improving maternal and child healthcare.

Consequently, the objective of the present study was to describe the clinical and epidemiological profile, management and neonatal prognosis of pregnant women with COVID-19 requiring respiratory support and receiving care at referral centers in northeastern Brazil.

## Methods

This was a descriptive study that included pregnant and postpartum women with COVID-19 who required respiratory support. The study was conducted between April and December 2021. It is an offshoot of a multicenter study, initiated in 2020 and registered at Clinical Trials under reference NCT04462367, which was conducted to describe the clinical, epidemiological and laboratory characteristics of cases of COVID-19-related maternal near miss and death in northeastern Brazil. Data were collected at six referral centers in northeastern Brazil: two in the state of Pernambuco, four in Paraíba and one in Ceará. The study involved both retrospective and prospective stages. Sample size was not calculated because all the patients who received care at the referral hospitals in the first two years of the pandemic who met the eligibility criteria were included in the study. A non-probabilistic, consecutive convenience sample was obtained. Patients whose hospital records could not be located or were incomplete were excluded from the current analysis.

The variables analyzed were: patients’ biological and sociodemographic characteristics (maternal age, skin color/ethnicity, education, maternal occupation including income, marital status and origin of the patient prior to admission); obstetric characteristics (whether pregnant or postpartum at inclusion in the study, number of pregnancies, parity, number of prenatal visits, gestational age at admission and mode of delivery), characteristics of respiratory rate and oxygen saturation; comorbidities/complications associated with pregnancy/postpartum (SARS, hypertensive syndromes, obesity/overweight and asthma), COVID-19-related symptoms (cough, fever, headache, dyspnea and chest pain) and radiologic findings (chest x-ray and tomography) and forms of treatment administered during hospitalization (admission to ICU, treatment with corticoids, antibiotics, neuromuscular blocking agents, assisted mechanical ventilation and non-invasive ventilation). Finally, maternal outcome (maternal near miss and death) and neonatal outcome (1-minute and 5-minute Apgar scores) were evaluated.

COVID-19 was confirmed by positive real-time polymerase chain reaction. SARS was defined as a flu-like syndrome (fever, cough, dyspnea and other non-specific symptoms) together with oxygen saturation (SpO_2_) <95%, respiratory distress or tachypnea, hypotension and worsening clinical conditions of the primary disease.^(8)^ Need for mechanical ventilation was defined as having used one of the following modes of treatment: non-invasive ventilation (nasal catheter, Venturi mask, non-rebreather mask, continuous positive airway pressure [CPAP]); mechanical ventilation; extracorporeal membrane oxygenation [ECMO]; assisted mechanical ventilation in prone position and spontaneous ventilation in prone position.

According to the definition of the World Health Organization (WHO), maternal near miss (MNM) refers to a woman who nearly died but survived a complication that occurred during pregnancy, childbirth or within 42 days of termination of pregnancy.^(9)^ Maternal death is defined as the death of a woman while pregnant or within 42 days of termination of pregnancy from any cause related to or aggravated by the pregnancy or its management, but not from accidental or incidental causes.^(9)^ Neonatal near miss refers to a newborn infant with a severe complication at birth (five-minute Apgar <7; birthweight <1,750 grams or gestational age <33 weeks) or who requires certain management (parenteral antibiotics, nasal CPAP or intubation up to 7 days and before the 28^th^ day of life, phototherapy within 24 hours of life, cardiopulmonary resuscitation, use of vasoactive drugs, use of anticonvulsants, use of blood derivatives, use of corticoid therapy to treat refractory hypoglycemia and surgery) but who survives up to the 27^th^ day of life.^(10)^ Neonatal death was defined as death occurring within the first 27 days of life.^(11)^

The statistical analysis was performed using the Epi Info software program, version 7.2.5 and Medcalc, version 20.112. Measures of central tendency and dispersion were used to describe the numerical variables, while frequency distribution tables were constructed for the categorical variables.

The institutional review board approved the study protocol under reference CAAE 58466822.0.0000.5201. Since the research consisted of analyzing a secondary database previously created for the original study, the need for informed consent was waived.

## Results

**Figure 1:**
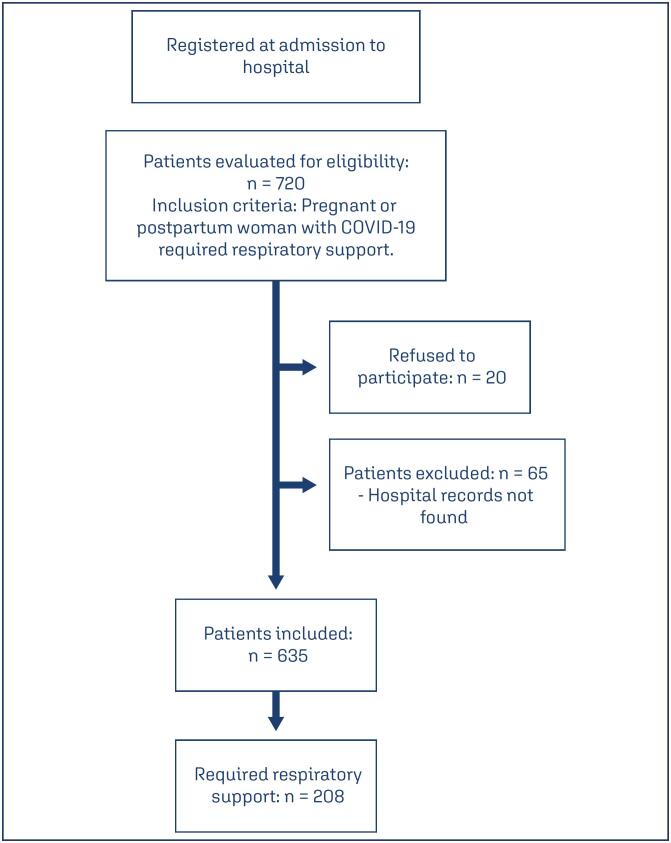
Patient admission process

During the period of the original study, 720 patients were admitted to hospital. Of these, 20 refused to participate. In addition, 65 were excluded from the current analysis because their records could not be located. Of the 635 remaining patients, 208 (32.7%) required some form of respiratory support and constituted the present sample (Figure 1).

The mean age of the participants was 28.9 ± 7.1 years. Most (52.8%) were brown-skinned, while 13.5% were white and 5.3% were black; however, in 27.4% of cases this information was missing from the records. In relation to their sociodemographic characteristics, 35 patients (31.3%) had little schooling and 39 (41.1%) had a personal income. Overall, 48 (23.1%) were married and 44 (21.2%) were single; however, in 77 cases (37%) information on marital status was missing from the charts. Most of the women (50.9%) were referred to this hospital from another healthcare institute, while 21.2% came straight from home (Table 1).

**Table 1:** Sociodemographic, clinical and obstetric characteristics of the pregnant women affected with COVID-19 who required respiratory support

Characteristics		
Age (years) (mean/standard deviation)		28.9(7.1)
Ethnicity/skin color (n / %)		
	White	28(13.5)
	Brown	110(52.8)
	Black	11(5.3)
	Indigenous	1(0.5)
	Of Asian descent	1(0.5)
	Unknown	57(27.4)
Poor education* (n / %)		35(31.3)
Income of her own** (n / %)		39(41.1)
Marital status (n / %)		
	Married	48(23.1)
	In a stable union	35(16.8)
	Divorced/separated	3(1.4)
	Widowed	1(0.5)
	Single	44(21.2)
	Unknown	77(37.0)
Origin of patient (n / %)		
	Came straight from home	44(21.2)
	Referred from a primary healthcare unit	15(7.2)
	Referred from another hospital	106(50.9)
	Unknown	43(20.7)
Pregnancies		
	Median/interquartile range	3(2-4)
	Range	1-9
Parity		
	Median/interquartile range	1(1-3)
	Range	0-8
Obesity or overweight described on hospital records*** (n / %)		46(36.8)
Hypertensive syndromes**** (n / %)		69(36.9)
Number of prenatal visits		
	Median/interquartile range	5(3-6)
	Range	0-9
Gestational age at admission (of the women admitted to hospital while pregnant) (in weeks)		
	Median/interquartile range	32.5(29-35.5)
	≤ 22 weeks (n / %)	16(8.8)
	23-28 weeks (n / %)	28(15.4)
	29-34 weeks (n / %)	60(33.0)
	35-36 weeks (n / %)	47(25.8)
	≥ 37 weeks (n / %)	31(17.0)
	Admitted postpartum (n / %)	26(12.5)

* Information available for 112 patients;

** Information available for 95 patients;

*** Information available for 125 patients;

**** Information available for 187 patients

Patients had had from 1 to 9 pregnancies, with a median of 3 (interquartile range [IQR] 2-4) and the number of births ranged from 0 to 8, with a median of 1 (IQR 1-3). The number of prenatal visits attended ranged from 0 to 9, with a median of 5 (IQR 3-6), while the median gestational age at admission was 32.5 weeks (IQR 29 - 35.5 weeks). When gestational age was stratified by week intervals, 60 patients (33%) were at 29-34 weeks of pregnancy, 47 (25.8%) were at 35-36 weeks and 31 patients (17%) were over 37 weeks pregnant. In 26 cases (12.5%), the patient was admitted to hospital during the postpartum period (Table 1).

At admission, 46 women (36.8%) were obese or overweight and 69 (36.9%) had hypertensive syndromes. Furthermore, 13.4% of the women had gestational diabetes and 6% had pre-gestational diabetes mellitus (Table 1).

Median respiratory rate at admission was 28 breaths/minute (IQR 22-32), with the worst median value during hospitalization being 34 breaths/minute (IQR 28-40). Median SpO_2_ at admission was 96% (IQR 93-98), with the worst median during hospital stay being 91% (IQR 88-94) (Table 2).

**Table 2:** Respiratory rate and oxygen saturation (SpO_2_) in the pregnant women with COVID-19 who required respiratory support

	At hospital admission		Worst value during hospitalization	
Respiratory rate (breaths/minute) (median / interquartile range)	28	22-32	34	28-40
Respiratory rate >25 breaths/minute (n / %)	111*	60	160**	85,5
SpO_2_ levels (%) (median /interquartile range)	96	93-98	91	88-94
SpO_2_levels <95% (n / %)	66***	34.4	149****	76.8

* Data available for 186 cases;

** Data available for 187 cases;

*** Data available for 192 cases;

**** Data available for 194 cases

The principal signs and symptoms in the patients were: headache (80.7%), dyspnea (78.8%), fever (65.8%) and dry cough (63.5%). The criteria for SARS were present in 168 patients (80.7%). While most patients were not submitted to imaging tests, abnormalities were found in 70/87 patients (80.5%) submitted to chest x-ray and in 32/46 (69.6%) patients who had a tomography. The most common abnormality found at x-ray was diffuse pulmonary infiltrate, present in 51.4% of the women evaluated, while the most common abnormality found at tomography was ground-glass opacity, found in 93.7% of patients evaluated (Table 3).

**Table 3:** Clinical and obstetric characteristics and neonatal outcomes for the pregnant women affected with COVID-19 who required respiratory support

Characteristics		n(%)
Dry cough		132(63.5)
Fever		137(65.8)
Headache		168(80.7)
Dyspnea		164(78.8)
Chest pain		28(13.5)
Criteria for SARS		168(80.7)
Abnormalities at chest x-ray*		70(80.5)
	Diffuse pulmonary infiltrate	36(51.4)
Abnormalities on computed tomography of the chest**		32(69.6)
	Ground-glass opacity	30(93.7)
Corticoid therapy		151(74.7)
Antibiotic treatment***		171(82.2)
Neuromuscular blocking agents****		34(16.3)
Admission to maternal ICU		150(76.1)
Mode of delivery****		
	Vaginal	13(8.4)
	Cesarean section	142(91.6)
1-minute Apgar score (median /interquartile range)		8(7-9)
5-minute Apgar score (median /interquartile range)		8(5-9)
Neonatal near miss*****		17(14.2)
Neonatal death*****		7(4.5)
Maternal near miss		50(24.0)
Maternal death		27(12.9)
Total		208(100)

* Performed in 87 patients;

** Performed in 46 patients;

*** Data available for 204 patients;

**** Data available for 120 cases, e Data available for 156 cases

A total of 151 patients (74.7%) were treated with corticoids for COVID-19, while 171 (82.2%) used antibiotics, 34 (16.3%) neuromuscular blocking agents and 150 (76.1%) were admitted to the ICU. There were 50 cases (24%) of maternal near miss and 27 (12.9%) women died (Table 3). Information on the mode of delivery was available for 155 patients, with 142 patients (91.6%) having undergone a cesarean section and 13 (8.4%) having had a vaginal delivery. In relation to the neonatal endpoints, the median 1-minute Apgar score was 8 (IQR 7-9) and the median 5-minute Apgar score was 8 (IQR 5-9). Adverse neonatal outcomes included 17 cases (14.2%) of neonatal near miss and 7 neonatal deaths (4.5%) (Table 3).

A total of 208 women required some type of ventilation strategy or respiratory support during hospitalization. Non-invasive ventilation was used in 186 cases (89.4%), with 115 using a nasal catheter, 26 (16.8%) a non-rebreather mask, 9 (4.3%) a Venturi mask and 7 (3.4%) CPAP. Invasive mechanical ventilation was the resource used in 78 cases (37.5%), with 1 patient using ECMO, 17 (8.2%) using spontaneous ventilation in the prone position and 10 (4.8%) assisted mechanical ventilation in the prone position (Table 4).

**Table 4:** Characteristics of the respiratory support required by the pregnant women with COVID-19

Characteristic		n(%)
Non-invasive ventilatory support		186(89.4)
	Nasal catheter	115(55.3)
	Venturi mask	9(4.3)
	Non-rebreather mask	26(16.8)
	Continuous positive airway pressure (CPAP)	7(3.4)
Mechanical ventilation		78(37.5)
	Extracorporeal membrane oxygenation (ECMO)	1(0.5)
	Assisted mechanical ventilation in prone position	10(4.8)
	Spontaneous ventilation in prone position	17(8.2)
Total		208(100)

The same patient could have used more than one method of ventilatory support

## Discussion

Although the acute phase of the COVID-19 pandemic has passed, its consequences on maternal and neonatal health remain a crucial field of study. Pregnant women are a vulnerable population, and our research provides valuable data on how COVID-19, specifically Severe Acute Respiratory Syndrome, affected this population in the Northeast of Brazil. The hospital records of 635 pregnant or postpartum women were analyzed and, of these, 208 were found to have required respiratory support. The sociodemographic characteristics of these patients show that most were brown-skinned, married and had been referred from other hospitals. The most common comorbidities present were obesity, diabetes and hypertensive syndromes.

The mean age of the patients who required ventilatory support in the present study was lower than that reported in a prospective, multicenter study involving pregnant women who had tested positive for Severe Acute Respiratory Syndrome Coronavirus 2 (SARS-CoV-2). In that study, published in April 2020 by the German Society of Perinatal Medicine, the median age of the pregnant women who required intensive care was 33 years.^(12,13)^ An epidemiological bulletin published in Brazil showed that, of the cases of SARS in pregnant women who required ventilatory support, the age group with the most number of notified cases of COVID-19 was the 20-29-year age group,^(14)^ as reported in the present study.

Most of the women in the present study self-reported as being brown-skinned, followed by white and then black women. This finding is in agreement with a survey of data from the Brazilian nationwide surveillance database on seasonal influenza, which described pregnant women of 10 to 49 years of age hospitalized due to SARS-CoV-2 between January and November 2020, in which almost half the sample self-reported as being brown-skinned.^(15)^ The inequalities associated with race/ethnicity are well known in Brazil. Evidence of this disproportional impact is also apparent in ethnic groups that have been historically oppressed in the country, the current epicenter of the global pandemic.^(16)^ In Brazil, the intersection of gender, race and social class compounds the tragedy of the maternal deaths resulting from COVID-19, particularly when the country fails to adopt measures that would truly contain pandemics.^(15)^ On the other hand, the large amount of missing data on skin color/race may in itself be a reflection of the structural racism in Brazilian society that permeates healthcare facilities.^(17)^

A significant proportion of the sample had little schooling. Socioeconomic status and poor education can accelerate worsening maternal health, since these factors affect the quality of prenatal care and the effectiveness of treatment, hence being associated with maternal morbidity and mortality.^(17)^ Furthermore, there are chronic problems associated with women's healthcare in Brazil, including insufficient resources, poor quality prenatal care, fewer hospital beds than are required, difficult access to healthcare facilities, racial disparities and obstetric violence.^(4)^

Most of the patients in the present study had been referred from another hospital, with the majority of the others coming straight from home. The possible explanation for the high rate of referral from other hospitals is that the data were extracted from referral centers for northeastern Brazil; therefore, many of the patients who were in a critical state had been transferred from other healthcare facilities. We failed to find data referring to other similar samples, thus making comparison with other databases impossible.

The presence of comorbidities increases the risk of developing more severe respiratory distress and introduces a greater likelihood of requiring hospitalization, of developing complications and of death.^18^ Part of the sample was described in the hospital records at admission as being obese or overweight. A study conducted in Brazil using data from the Brazilian nationwide surveillance database on seasonal influenza described 6,073 pregnant women with COVID-19 between 2020 and 2021 and highlighted obesity as one of the most common comorbidities in that population.^(18)^ Some parameters could explain the greater frequency of respiratory complications in obese patients, including: reduced functional residual capacity and expiratory residual volume, as well as hypoxia and ventilation/perfusion abnormalities.

Hypertensive syndromes and gestational diabetes were also very common in the present study. The number of cases reported here is higher than numbers from a systematic review that analyzed 441 pregnant women with COVID-19 in 16 countries and identified the presence of hypertensive diseases (9%) and diabetes (11%) as being among the most common comorbidities.^(19)^

We believe that the high rate of pregnant women with chronic systemic arterial hypertension and also preeclampsia is due to the peculiarities of the hospitals included in the study. All are tertiary hospitals that, during the pandemic, acted as referral services for high-risk pregnancy. Since all pregnant women were tested for COVID-19 at admission, consequently the number of positive tests in women with such comorbidities was higher. On the other hand, the association between COVID-19 and preeclampsia has already been shown numerous times in other studies, with prognosis in such cases being poorer.^(19,20)^ Our research group is also investigating this association; however, it was not one of the objectives of the present study.

Median gestational age was 32.5 weeks. Due to the increase in the size of the uterus in the third trimester of pregnancy, the movement of the diaphragm is restricted, reducing total lung capacity. A study conducted in Brazil found a higher frequency of COVID-19-related SARS in women in the third trimester of pregnancy,^(7)^ corroborating data published by the Ministry of Health in 2021^(21)^ and the present results.

The number of prenatal visits ranged from 0 to 9, with a median of 5. One of the reasons for this insufficient number of prenatal visits could be the fact that the women began prenatal care late, reflecting difficulties in obtaining confirmation of pregnancy. This clearly highlights the need to improve women's access to diagnostic tools and the need to direct them towards prenatal care at an earlier stage.^(22)^ On the other hand, it is possible that the percentage of patients admitted at a lower gestational age could explain the lower median number of visits. Furthermore, information on prenatal visits was missing from a proportion of the sample, hampering analysis.

Although COVID-19 may affect several different organs and systems, SARS-CoV-2 is particularly aggressive to the respiratory system, leading to a wide array of symptoms ranging from a common cold to severe respiratory distress.^(23)^ Median respiratory rate at admission was 28 breaths/minute, with the worst median value during hospital stay being 34 breaths/minute. Median SpO_2_ at admission was 96%, with the poorest median value during hospitalization being 91%. The increased inflammation and reduced innate immune response may provide answers regarding the increased susceptibility to severe SARS-CoV-2 disease in patients with these conditions, including pregnant women.^(24)^ An exploratory cohort study conducted in Brazil on COVID-19 in pregnancy identified the risk factors leading to a need for oxygen in pregnant and postpartum women with COVID-19. The study revealed a greater risk of requiring oxygen therapy in patients with a respiratory rate of 24 breaths/minute and SpO_2_ <95%, data similar to those found in the present study.^(25)^

The principal signs and symptoms found in this group of pregnant women were: headache, dyspnea, fever and dry cough. Similar data were reported from a series of cases investigated in Iran.^(26)^

Although imaging tests were only performed in part of the sample, results showed a high frequency of abnormalities. A systematic review involving 1,316 pregnant women with confirmed COVID-19 reported the most common findings at computed tomography as ground-glass opacity followed by bilateral pneumonia, with prevalence rates of 65.8% and 57.9%, respectively.^(27)^ The frequency of findings at tomography in the present sample is higher than that reported in the systematic review, which could be explained by the fact that the present study included a subpopulation of more severely ill patients among those with the disease, defined as such by the fact that they required ventilatory support. Furthermore, at the specific healthcare units involved in this study, imaging tests are not routinely requested but are restricted to the most complicated cases, which may have resulted in a selection of patients in whom abnormalities at x-ray and tomography were more likely to be found.

In the present study, 74.7% of the pregnant women were given corticoids for the treatment of COVID-19. According to the Ministry of Health guidelines, corticoids should be considered seven days after the onset of symptoms if the patient still has significant lung involvement.^(28)^

In the present study, the mode of delivery was recorded for 155 cases, with most having undergone cesarean section. This finding raises the question of what is taken into account when indicating the mode of delivery, since high rates of cesarean section have also been reported in several other studies, including a systematic review involving 385 cases of COVID-19 in pregnancy. In that review, 252 births were reported, with 175 cesarean sections (69.4%) and 77 vaginal deliveries (30.6%).^(29)^ Since the present study is restricted to describing the fraction of patients who required ventilatory support, the limited number of vaginal deliveries can probably be explained by the clinical severity of the patients evaluated here.

Analysis of severe maternal outcomes showed that 24% of the women experienced maternal near miss and 12.9% died. In this respect, a study conducted in the city of New York in the United States reported a maternal mortality rate of 15% and a mean duration of ICU stay due to COVID-19 of 8 days.^(30)^ Another study conducted in Brazil investigated the characteristics and outcome of pregnant women with a SARS-CoV-2 infection and other severe acute respiratory infections (SARI) in Brazil. Mortality among these pregnant women with COVID-19 was high compared to that of other groups with SARI.^(7)^

In relation to the neonatal outcomes, the median 1-minute and 5-minute Apgar scores were 8. This finding is similar to data from one of the first retrospective analyses conducted in China in 2020 involving 9 liveborn infants, with a 1-minute Apgar score of 8-9 and a 5-minute Apgar score of 9-10.^(31)^ In relation to the more severe outcomes, neonatal near miss was recorded in 22 cases and there were 7 neonatal deaths. A systematic review evaluated data on studies from ten countries and showed that, of the 256 newborn infants analyzed, the outcomes reported included admission to an ICU in 8 cases (3.1%), neonatal mechanical ventilation in 3 (1.2%), newborn respiratory distress syndrome in 12 (4.7%), neonatal pneumonia in 3 (1.2%) and disseminated intravascular coagulation in 3 (1.2%). Three infants died.^(32)^

The principles of managing COVID-19 during pregnancy include early isolation, infection control procedures, oxygen therapy, prevention of fluid overload, empirical control with antibiotics (secondary to the risk of bacterial infection), monitoring for uterine contractions, early mechanical ventilation in cases of progressive respiratory failure, an individualized birth plan, and clinical care from a multidisciplinary team.^(33)^ In the present study, non-invasive ventilatory support was the principal resource used, with the most common method being a nasal catheter in slightly over half the patients, followed by a non-rebreather mask.

The objective of ventilatory support is to maintain the levels of maternal partial pressure of oxygen in arterial blood (PaO_2_) at 65-70 mmHg or higher to guarantee adequate fetal oxygen supply. This is contrary to the management for non-pregnant women in whom low levels of PaO_2_ are acceptable.^(34)^ A systematic review evaluated the effects of coronavirus on pregnant women and found that around 78.8% were treated with oxygen therapy, while 18.1% required mechanical ventilation.^(27)^

A considerable number of the patients in the present study were admitted to the maternal ICU and mechanical ventilation was used as a resource in 37.5% of these cases. This finding emphasizes the high numbers in Brazil compared to other countries such as the United States.^(22)^ A cohort study conducted in the United States compared the clinical characteristics and outcomes of hospitalized women with and without COVID-19. The rate of American women with COVID-19 who required intensive care was 3.3%, whereas only 1.3% of patients in that study required mechanical ventilation.^(22)^

Compared to other epidemics of flu-like syndromes, the rate of admission to an ICU for women with COVID-19 (19.5% of cases) was higher than that for SARI of unknown etiology (16.8%) or for influenza (15.8%). The present findings suggest that adverse outcomes in pregnancy during the COVID-19 pandemic period could also be related to poor quality obstetric care, social factors and difficulty in accessing healthcare.^(7)^ The actions of the Brazilian government to contain the COVID-19 pandemic disregarded global recommendations, failing to emphasize the need for social isolation or provide universal screening, with disastrous results. COVID-19 remains an active field of study, with new variants and potential new waves of infection. Our study contributes critical information to the scientific literature by detailing the risk factors and clinical course of SARS in pregnant women infected with SARS-CoV-2.

The limitations associated with the present study include the fact that the data were obtained from analyzing hospital registries, with records for different variables being missing from some charts. Therefore, much information was unknown. In Brazil, the quality of registering healthcare procedures often varies between metropolitan and non-metropolitan areas of the country, hampering access to data on hospitalizations. On the other hand, the strongpoints of the study include the number of patients evaluated and their specific characteristics. The data collected and the conclusions of our study can also serve as a basis for future research exploring the long-term sequelae of COVID-19 in pregnant women and their newborns, in addition to being useful for comparison with data from future pandemics or severe respiratory conditions that may arise. Although the study was conducted in Brazil, its conclusions have global implications, given that COVID-19 affected pregnant women worldwide. Publishing this article will expand the reach of the findings, allowing other regions and countries to learn from the experience of Northeast Brazil.

## Conclusion

The most common characteristics of the patients with COVID-19 who required respiratory support included being brown-skinned, having little schooling, poor socioeconomic conditions and having been referred from another healthcare unit. The most common symptoms were headache, dyspnea, fever and dry cough. More severe respiratory findings such as respiratory distress and SpO_2_ <95% were also common in this population. Non-invasive ventilation was used in the majority of cases, followed by mechanical ventilation. Adverse obstetric outcomes were common, notably the high rate of cesarean section, presence of criteria for SARS, and COVID-19-related maternal near miss and death. Further studies should be conducted to delve deeper into the issues raised in this sample.
